# Selective Oxidation of Cellulose—A Multitask Platform with Significant Environmental Impact

**DOI:** 10.3390/ma15145076

**Published:** 2022-07-21

**Authors:** Ioana A. Duceac, Fulga Tanasa, Sergiu Coseri

**Affiliations:** Department of Polyaddition and Photochemistry, “Petru Poni” Institute of Macromolecular Chemistry, 700487 Iasi, Romania; iaduceac@gmail.com (I.A.D.); coseris@icmpp.ro (S.C.)

**Keywords:** cellulose, selective oxidation, materials for environmental applications

## Abstract

Raw cellulose, or even agro-industrial waste, have been extensively used for environmental applications, namely industrial water decontamination, due to their effectiveness, availability, and low production cost. This was a response to the increasing societal demand for fresh water, which made the purification of wastewater one of the major research issue for both academic and industrial R&D communities. Cellulose has undergone various derivatization reactions in order to change the cellulose surface charge density, a prerequisite condition to delaminate fibers down to nanometric fibrils through a low-energy process, and to obtain products with various structures and properties able to undergo further processing. Selective oxidation of cellulose, one of the most important methods of chemical modification, turned out to be a multitask platform to obtain new high-performance, versatile, cellulose-based materials, with many other applications aside from the environmental ones: in biomedical engineering and healthcare, energy storage, barrier and sensing applications, food packaging, etc. Various methods of selective oxidation have been studied, but among these, (2,2,6,6-tetramethylpiperidin-1-yl)oxyl) (TEMPO)-mediated and periodate oxidation reactions have attracted more interest due to their enhanced regioselectivity, high yield and degree of substitution, mild conditions, and the possibility to further process the selectively oxidized cellulose into new materials with more complex formulations. This study systematically presents the main methods commonly used for the selective oxidation of cellulose and provides a survey of the most recent reports on the environmental applications of oxidized cellulose, such as the removal of heavy metals, dyes, and other organic pollutants from the wastewater.

## 1. Introduction

In recent decades, the field of green materials has recorded a steady development in terms of design, production and use of a wide variety of materials from renewable resources. Their diversity derives from their nature, structure and specific properties, type of processing, intended applications, etc. Even more, the range of applications is constantly expanding due to innovative approaches (such as, the preparation of multicomponent systems with supramolecular multilevel architectures) which transform materials already in use into new ones by additivation (addition of compatibilizing agents, emulsifiers, UV-vis absorbing fillers, ion exchangers, etc.), hybridization (use of components of the same nature and different processing, i.e., fabrics made of woven, non-woven and knitted mats, or of the same processing but of different nature, i.e., textile fibers interlaced with metal threads), further processing and post-processing.

Theoretical and practical improvements in the use of cellulose and its derivatives in the manufacture of new and high-performance materials have highlighted two primary issues: (a) when it comes to imparting significantly improved bulk properties (or even new ones) to materials in a highly effective manner, using nanostructured cellulose (nano-fibers, nano-crystals, nano-whiskers) is a wise choice; (b) chemical reactions (i.e., oxidation) applied to functionalize cellulose have proved to be a useful tool not only for expanding the range of applications, but also for boosting the specificity and lowering the negative environmental impact [1,2,3].

In recent decades, social and economic factors, such as overpopulation of urban areas, aggressive industrialization, large-scale chemicalization of the food chain and hyper-processing of food, have entailed an intense degradation of the quality of drinking water due to the presence of high amounts of organic and inorganic pollutants (petroleum, heavy metals, dyes, pharmaceuticals, organic solvents, manure, synthetic fertilizers, insecticides and herbicides, etc.) [4,5,6,7]. Even more, the water resources, either ground water or surface ones, are seriously decreasing at planetary scale due to the dramatic climate changes we have been witnessing in the last century. In result, the increasing societal demand for freshwater made the purification of wastewater one of the major research issue for both academic and industrial R&D communities [8,9,10,11,12]. This issue has been acknowledged by the United Nations General Assembly as one the goals of the sustainable development envisaged in the 2030 Agenda for Sustainable Development [13]. The wastewater purification is a part of the strategy designed to allow a sustainable management of water (recovery, recycle, reuse) and to enable facile access to clean water for people [14]. The challenges in the treatment of the industrial water refer not only to the limitation of the environmental hazard [15,16,17], but to find suitable methods and materials having a high specificity and effectiveness for active/reactive purification. Currently, the treatment of industrial wastewater has been approached from different angles (i.e., application of nanotechnology in water purification [18]; employ of metal organic frameworks (MOFs) [19]; use of ultrasounds technology [9]; membraneless approach [20]), which yielded in numerous valid experimental protocols, some of them fit for the technology transfer, and separation media. There is a wide variety of materials employed in wastewater purification (membranes, foams, gels, nonwoven mats, particles and fibers of micro- and nanometer scale) ranging from raw and functionalized natural polymers [21,22,23] to synthetic and mixed polymer membranes [24,25], composite materials [26,27,28,29], or even membranes [30,31,32] and nanoparticles of different nature [33,34,35].

Because of their effectiveness, availability, and low manufacturing cost, raw cellulose and even agro-industrial waste have been considered for environmental applications, such as decontamination of industrial water [36,37,38,39,40]. Oxidation is a reliable method for cellulose functionalization because it yields in products with various structures and properties depending on the substrate, reagents, reaction parameters, and medium. It was also used to change the cellulose surface charge density, a prerequisite condition to delaminate fibers down to nanometric fibrils through a low-energy process [41].

Selective oxidation of cellulose, one of the most important methods of chemical modification, turned out to be a platform to obtain new high performance, versatile, cellulose-based materials, having many other applications aside the environmental ones: in biomedical engineering and healthcare [42,43,44,45,46], energy and smart materials [47,48,49,50,51], barrier applications [52,53,54], active food packaging [55,56,57,58]. Various methods of oxidation of carbohydrate polymers have been studied, but in the case of neutral polysaccharides (starch, cellulose) these processes evolved with low specificity and reduced yields. Even more, under more aggressive conditions (strong oxidants such as hypochlorite, periodate or nitric acid) [59], all hydroxylic groups, either primary or secondary, have been oxidized. Oxidation with nitrogen dioxide (N_2_O_4_) or nitrite/nitrate in concentrated phosphoric acid caused depolymerization side reactions, although the presence of phosphoric acid limited the degradation of the macromolecular chain up to a certain degree [60]. Recently, the oxidation was performed in high-pressure CO_2_ which allowed not only an improved yield, but a better purification of the final products as well [61]. Oxidation of polysaccharides under milder conditions took place with very low oxidation yields [60].

In contrast, oxidation in the presence of TEMPO (2,2,6,6-tetramethylpiperidin-1-yl)oxyl) proved to be of interest due to its highly increased selectivity, short reaction time, mild reaction conditions and limited side reactions [60]. Even more, the method allowed a significant number of C6 hydroxyl groups to be transformed into carboxylate moieties, while preserving the original crystallinity and crystal width when applied to wood celluloses [62]. Another method of selective oxidation of polysaccharides is periodate oxidation [63]. The reaction system is able to cleave the C2–C3 bonds in monomer units of cellulose and oxidize the newly formed vicinal hydroxyl groups into 2,3 dialdehyde moieties. An advantage of this method is the possibility to further oxidize the dialdehyde groups to carboxylic ones. Combining TEMPO and periodate methods into one single protocol, it was possible to obtain highly carboxylated (2,3,6-tricarboxylate) cellulose, with an increased substitution degree and preserved crystallinity [64]. Many studies have reported on the functional products of cellulose oxidation, but TEMPO-mediated and periodate oxidation reactions stood out due to their enhanced regioselectivity, high degree of substitution, mild conditions, increased yields. Even more, oxidized cellulose thus obtained can be further processed into more complex materials [64,65,66,67,68,69,70,71,72,73].

This study systematically reviews the main methods for the selective oxidation of cellulose and provides a survey of the most recent reports on the environmental applications of oxidized cellulose, including the removal of heavy metals, dyes, and other organic and inorganic pollutants from the wastewater.

## 2. Selective Oxidation of Cellulose by Different Methods

Cellulose is one of the most chemically and mechanically stable neutral polysaccharides. This is due to its tightly linear orientation, which results in a stiff rod-like conformation, to which the equatorial conformation of the β(1→4)-glycosidic bonds contributes, to the extensive hydrogen bonding that occurs between the macromolecular chains, and to its high crystallinity.

Nevertheless, cellulose is still prone to undergo derivatization processes due to its chemical structure that comprises numerous hydroxylic groups, both primary and secondary. Therefore, reactions such as esterification, etherification, silanization and oxidation have been successfully employed in order to obtain cellulose fibers (of micro- and nanometric dimensions) with enhanced reactivity due to their enriched functionality: ester groups (carboxylate, sulphate, and phosphate esters), silane moieties, ether bridges, aldehydes, and carboxylic acids/salts [74].

Non-selective oxidation of cellulose can be performed in a wide variety of oxidative systems, in the presence of nitrogen oxides [75,76], nitrates and nitrites [77], peroxides [78], sodium chlorite [79], permanganates [80,81], and even ozone [82,83] or lead (IV) tetraacetate [77] as non-specific oxidants. Most of these reactions occur under severe conditions (aggressive pH, high pressure, elevated temperature) and entail chemical degradation reactions (depolymerization) along with the synthesis of functionalized cellulose. Even modern approaches turned out to have some limitations. Thus, employing supercritical CO_2_ as solvent, which allows oxidation to take place in a heterogeneous fluid-solid system where the contact area between substrate and reagents is highly enlarged, caused the oxidant reaction with the solvent and this partially inhibited its reactivity toward cellulose, yielding in a soft and homogeneous oxidation [61].

When it comes to selective oxidation, cellulose is extremely sensitive to reaction conditions (nature and type of oxidizing agent, pH of reaction medium, temperature, solvent, and so on), to the point where the final products differ not only in the nature of newly acquired functional groups, but also in the position in which they are linked. The following transformations occur depending on the type of hydroxyl group: aldehyde or carboxyl moieties are formed from primary hydroxyl groups (C6) [62], while the secondary ones (C2, C3) turn into ketone and aldehyde groups with the simultaneous opening of the glucose ring by the cleavage of the C2-C3 bond [63]. Selective oxidizing reagents extensively used for cellulose are nitroxyl radicals, either stable or non-persistent [59,84,85,86], and sodium periodate [63,87,88].

### 2.1. TEMPO-Assisted Oxidation (Stable Nitroxyl Radicals)

TEMPO-assisted oxidation of cellulose is a method that employs the stable nitroxyl radicals as selective oxidants. The reaction is highly regioselective, it takes place at the primary alcohol groups at C6 in anhydroglucose unit, and transforms them into carboxylic acid/salt moieties (polyuronic analogues). This process evolves with high yields and conversion degrees, although some depolymerization reactions are still present [60,89,90,91]. At the same time, it preserves the crystal width and most of the initial crystallinity of cellulose.

**Scheme 1:**
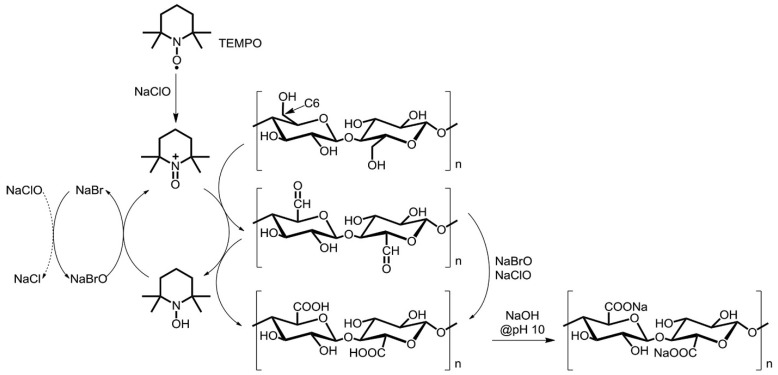
Mechanism of selective oxidation of cellulose in the presence of TEMPO and sodium hypochlorite (re-drawn after ref. [92]).

The oxidant species is the nitrosonium cation, generated *in situ*, in the first stage of the process, by the reaction of 2,2,6,6-tetramethylpiperidine-*N*-oxyl (TEMPO) with an oxidizing system (bromide salts react with sodium hypochlorite, yielding in hypobromide anions which further oxidize TEMPO). During the cellulose oxidation by the nitrosonium cation, this intermediate is reduced to *N*-hydroxy-2,2,6,6-tetramethylpiperidine, the reduced form of TEMPO. The entire process takes place at pH = 10.5, provided by the presence of NaOH in the reaction medium, which is necessary to neutralize the carboxylic acid moieties formed by oxidation. The mechanism of the TEMPO-assisted oxidation of cellulose is presented in Scheme 1.

The intermediate species presented in Scheme 1 are, as follows: A—*N*-hydroxy-2,2,6,6-tetramethylpiperidine (reduced form of TEMPO); B—nitrosonium radical (oxidized form of TEMPO); C—nitrosonium cation (active oxidant species for cellulose); D—sodium salt of the aldehyde hydrate. Thus, during the first stages of cellulose oxidation, primary hydroxyl groups were transformed into aldehyde groups, but hemiacetals moieties have been formed as well by intra- or intermolecular reactions between hydroxyl and aldehyde groups [93]. Aldehydes can turn into aldehyde hydrate sodium salts (intermediate D) and this reaction can explain the consumption of NaOH in amounts higher than the newly formed carboxylic acid groups [93]. In the final stage, aldehyde hydrates and/or hemiacetals are oxidized by the oxoammonium cations, when sodium salts of the carboxylic acid moieties resulted along with the reduced form of TEMPO (intermediate A). The oxidation cycle can be resumed by the formation of new nitroxyl radicals and oxoammonium cations, respectively.

It was demonstrated by theoretical calculations that this method of oxidation is highly selective under alkaline conditions, when the formation of a nitrosonium cation is much more probable than the formation of a peroxide complex [94].

This oxidation system TEMPO–NaBr–NaClO is pH- and temperature-sensitive. When the reaction was conducted at 4 °C, the lowest amount of amorphous cellulose has been reported to be depolymerized [95], but oxidation at room temperature allowed an increased number of carboxylic groups and limited the degradation reactions [96,97]. Significant depolymerization has been reported when pH was 11 [98], which may be due to hydroxyl radicals formed by the reaction of 2,2,6,6-tetramethylpiperidine with sodium hypobromide.

An alternative TEMPO-mediated oxidation system was designed and tested in order to further limit the depolymerization of cellulose. It employed a TEMPO derivative, namely 4-acetamido-TEMPO, sodium chlorite and hypochlorite (NaClO_2_ and NaClO), and the reaction was conducted at 40–60 °C and pH = 4.8–6.8, for 1–5 days [96,99]. In this system, the sodium chlorite was the primary oxidant, while the hypochlorite was added in catalytic amounts and initiated the oxidation of 4-acetamido-TEMPO to the corresponding N-oxoammonium cation.

A rather modern approach is the employ of electrochemical methods of oxidation [100]. The 4-acetamido-TEMPO-assisted electro-organic oxidation of cellulose takes place at room temperature, in the presence of phosphate buffer (0.1 M), when the reaction is conducted at pH = 6.8 for 45 h, without the contribution of NaClO_2_ or NaClO [101]. The oxidizing system allowed the functionalization of significant amounts of regenerated cellulose with the formation of carboxylate and aldehyde groups, while maintaining the initial morphology and without a relevant weight loss.

Holocelluloses from different sources have been selectively oxidized by employing two TEMPO-mediated systems, namely TEMPO/NaBr/NaClO and TEMPO/NaClO/NaClO_2_, which evolved in water, at pH 10 and 6.8, respectively [102]. The system working at pH = 10 afforded hollocelluloses with higher content in carboxylate groups, while the one working at pH = 6.8 allowed higher weight recovery ratios.

The TEMPO-mediated oxidation proved to be effective even for cellulose from different sources, such as sisal fibers when nanowhiskers have been successfully obtained [103], *Oryza sativa* that afforded nanofibers [104], or *Eucalyptus globus* that yielded in nanofibrils with reduced crystallinity [105], as well as cotton linters and ramie cellulose [106].

Some of the initial hydroxyl groups in cellulose that undergo TEMPO-mediated oxidation do not progress up to the ultimate step of oxidation, when carboxylate groups are produced, but remain partly oxidized to aldehydes. In order to convert these groups into carboxylates and, hence, increase the total carboxylate content, a post-oxidation step is required. It was reported that in the post-oxidation stage, the TEMPO-assisted oxidized cellulose was further treated with a solution of sodium chlorite (NaClO_2_, 25% *w*/*w*) and acetic acid, at pH = 3–4, and allowed to react at 40 °C, for 2 h [107]. The study found that following the second oxidation step, the carboxylate content increased significantly, as shown by calculations and low contact angle values for the produced samples.

Another reaction of selective oxidation of cellulose in the presence of stable nitroxyl radicals, that yielded in water soluble sodium salts of cellulose bearing carboxylic acid groups in positions C2, C3, and C6 (NaTCC), has been reported to take places when regenerated cellulose (82%) was treated with catalytic amounts of 2-aza-adamantane *N*-oxyl (AZADO) in water, under alkaline conditions [108]. AZADO is a stable nitroxyl radical able to oxidize the primary and secondary hydroxylic groups to carboxyl and carbonyl ones, respectively, in aqueous medium [109,110]. Working with the reaction system AZADO/NaBr/NaOCl in water, at room temperature and under alkaline conditions, it was possible to obtain NaTCC having an almost homogeneous structure through a one-step procedure. Excess of NaOCl and longer reaction time intervals have contributed to the success of the synthesis. It has been also proven that this reaction is difficult to apply to native cellulose.

### 2.2. Non-Persistent (Transient) Nitroxyl Radicals, Reactive Intermediaries for the Selective Oxidation of Cellulose

**Scheme 2:**
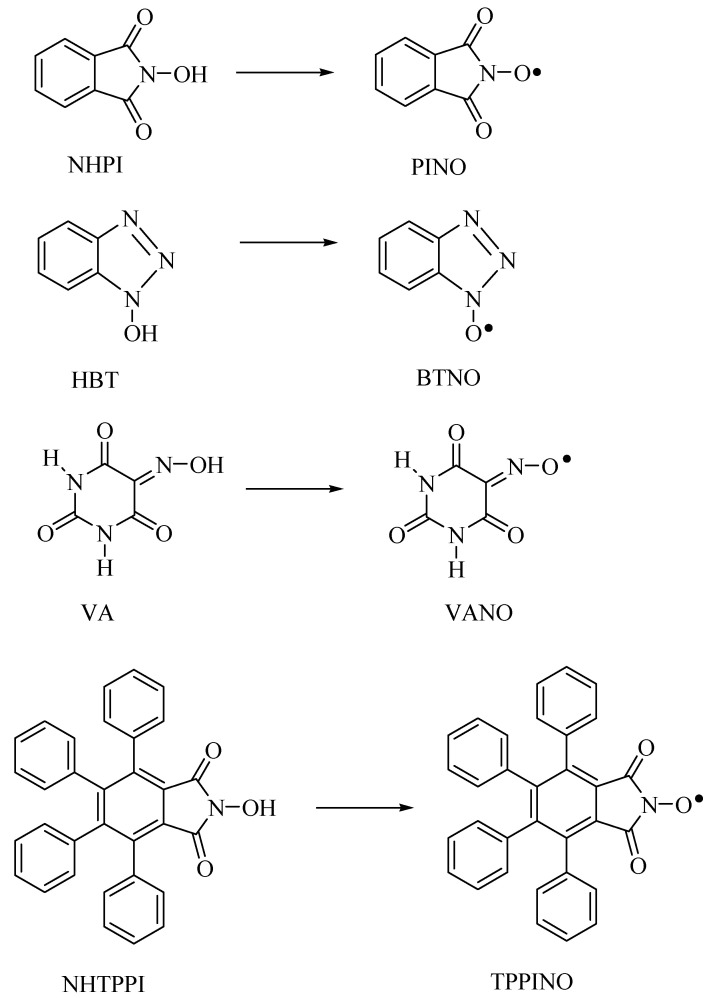
Structures of the precursors and their corresponding nitroxyl radicals (reprinted with permission from ref. [87]. Copyright year 2022, copyright owner’s name Elsevier).

Transient, non-persistent nitroxyl radicals have been successfully employed in the selective oxidation of cellulose since their first report back in 2009 [111]. Their major features include their formation *in situ* by the initiation (oxidation) of a corresponding precursor, present in the reaction medium in catalytic amounts, and the ability to restart the cycle following the reduction to the original form [68,71,87,112,113,114,115]. Aside *N*-hydroxyphthalimide (NHPI), which is the most widely used, other precursors have been studied, namely *N*-hydroxybenzotriazole (HBT), violuric acid (VA), and *N*-hydroxy-3,4,5,6-tetraphenylphthalimide (NHTPPI). Their structure and corresponding nitroxyl radicals are illustrated in Scheme 2.

**Scheme 3:**
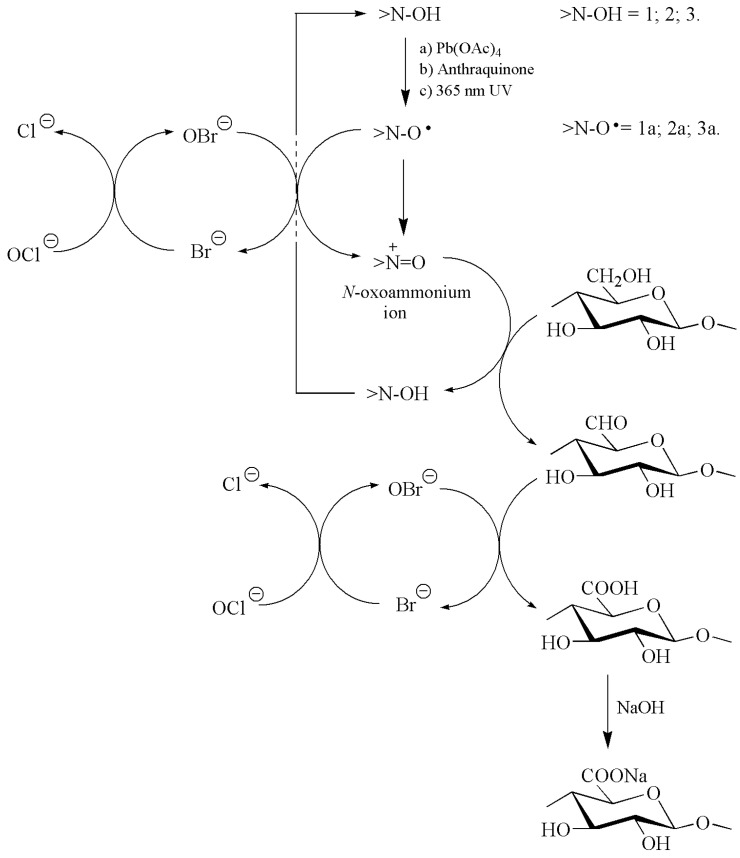
Mechanism of selective oxidation of cellulose in the presence of non-persistent nitroxyl radicals (reprinted with permission from ref. [87]. Copyright year 2022, copyright owner’s name Elsevier).

These mild oxidation systems have been specifically designed for the selective oxidation of viscose and cellulose [111,116], and the *in situ* generation of the reactive species occurred in the presence of various co-catalysts, such as lead tetraacetate, cerium (IV) ammonium nitrate, anthraquinone, and small amounts of sodium hypochlorite and bromide. The mechanism of oxidation in the presence of non-persistent nitroxyl radicals is illustrated in Scheme 3.

The formation of reactive species occurred in the first stage, by the means of the oxidation of precursor in the presence of the oxidizing system NaClO/NaBr, when nitroxyl radicals were formed and subsequently turned into *N*-oxoammonium cations. Then, the primary hydroxyl groups were oxidized first to aldehydes, and then to carboxylic moieties and carboxylates, respectively, due to the presence of NaOH in the reaction medium. Finally, the precursor is reformed by the reduction of the *N*-oxoammonium cations and the reactions cycle can be restarted.

Other reaction systems have been designed in order to limit or even eliminate the use of large amounts of NaBr required for the precursors regeneration and to re-initiate the cycle of reactions. Thus, a bromide-free experimental protocol was designed for the TEMPO-assisted oxidation of starch and methyl α-D-glucopyranoside [117]. For cellulose, a bromide-free protocol has been designed following the conditions of the aerobic oxidation of hydroxyl groups in the presence of CuCl_2_ in catalytic amounts [116]. In this case, Cu (II) cations were involved both in the conversion of the precursor NHPI to PINO radical and in its oxidation to PINO^+^ cation, the reactive oxidant of cellulose. The reduction of Cu(II) to Cu(I) allowed the oxidation of NHPI in a new cycle.

The selective oxidation of viscose and modal fibers was performed using another reaction system based on NHPI and molecular oxygen (in fact, dioxygen, also known as triplet dioxygen-^3^O_2_, the most stable and common allotrope of oxygen in air [118]), evolving at neutral pH and room temperature. It has been successfully tested, and only a slight decrease in the degree of polymerization and molecular weight of the initial cellulose has been reported [119]. The *in situ* formed PINO radical was able to abstract a hydrogen atom from the primary hydroxyl group, and the cellulose carbon-centered macroradical that was created reacted with oxygen generating the carboxylic acid moiety *via* the carbon-centered peroxyl radical.

### 2.3. Cellulose Selective Oxidation in the Presence of Sodium Periodate

**Scheme 4:**
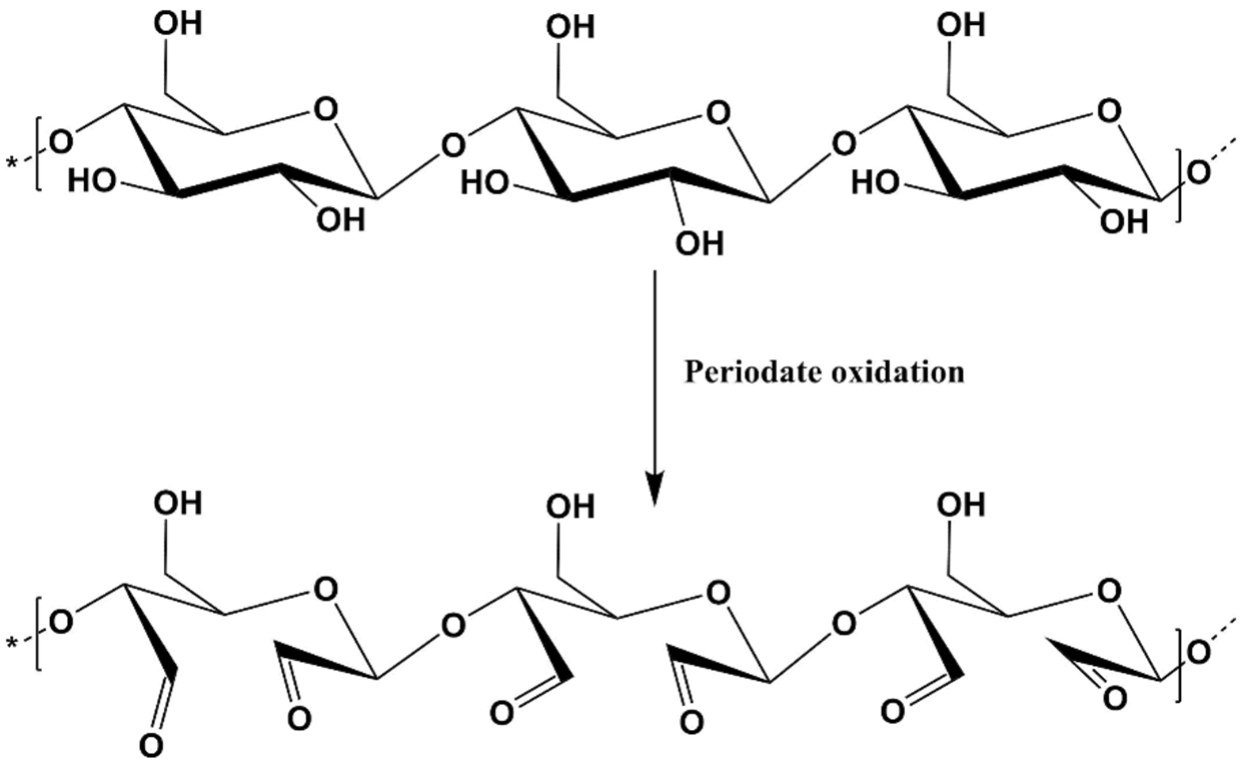
Mechanism of selective oxidation of cellulose in the presence of sodium periodate under mild conditions.

Sodium periodate is a highly selective cellulose oxidant as it is able to attack the vicinal secondary hydroxyl groups at C2 and C3 and cleave the C2-C3 bond in the anhydroglucose structural unit, resulting in two vicinal aldehyde groups (dialdehyde cellulose, DAC). The process evolves at acidic pH (≈3), at temperatures near to room temperature (20 °C), in the dark, for 24 ÷ 250 h [63]. The mechanism of selective oxidation of cellulose in the presence of sodium periodate under mild conditions is presented in Scheme 4.

Among the advantages of this method is the possibility to easily prepare cellulose nanofibrils with excellent dispersibility in high yields [120], and to further convert the aldehyde groups into carboxylic ones [121,122,123], primary alcohols [124], or imines (Schiff bases) using amines [121,125,126]. However, the high crystallinity of cellulose and the hemiacetal formation limited its application on large scale. Unlike the TEMPO-assisted oxidation, the periodate oxidation leads to DAC with a reduced crystallinity (depending on the degree of oxidation), but the microfibril shape is preserved [63]. Furthermore, the SEM analysis revealed that oxidized cellulose fibrils are bent, indicating a reduced stiffness.

According to earlier studies, the introduction of aldehyde groups in regions of high molecular weight is a distinct feature of periodate oxidation, and it was concluded that the periodate anion attacked the C2-C3 bonds in the crystalline regions since the early stages of the reaction, thus affecting the highly ordered alignment of macromolecules. Even when the overall degree of oxidation is relatively modest, the intermolecular hemiacetal bonds that occurred in regions with high degrees of oxidation contributed to the formation of more compact supramolecular structures [127].

In order to reduce the reaction time, the reaction was conducted at higher temperatures (85 °C), above the periodate decomposition temperature (55 °C), and in the presence of metal salts (LiCl, ZnCl_2_, CaCl_2_) as cellulose activators [88]. Under these conditions, it was possible to obtain DAC with a high content of aldehyde groups using lower amounts of periodate. Another method of optimization addressed two drawbacks, the slow kinetics and the dilute conditions, which were overcome by performing the periodate oxidation at high cellulosic pulp consistency with a weight ratio cellulose:water = 1:4 [128]. The experimental protocol consisted of two stages: first, the periodate, cellulose and water were vigorously ball milled for 2 min; the second stage lasted for 8 h, no further mixing was required and the system was allowed to rest and react. Thus, DAC with finely tuned degree of oxidation and high content of aldehyde groups was efficiently obtained.

A research on the periodate oxidation of two cellulose allomorphs, cellulose I and cellulose II, as well as their mixture, found that cellulose I oxidizes at the slowest rate, but cellulose II oxidizes much faster, likely due to its decreased crystallinity and an allomorph effect [129]. The thermogravimetric analysis indicated for all oxidized samples a decrease of the onset temperature, a slower thermal decomposition, and an enhanced stability above 350 °C.

In order to assess the dialdehyde content of DAC by viscometric measurements, cellulose oxidized in the presence of periodate was submitted to alkaline hydrolysis at room temperature, in both homogeneous (cupriethylenediamine) and heterogeneous (sodium hydroxide) system [97]. The method proved to be very sensitive to low degrees of oxidation. Topochemistry investigations on periodate oxidation of cellulose indicated that the periodate anion attack was fast but limited, and occurred in the amorphous regions of cellulose, thus causing a depolymerization of the macromolecular chains [130]. Kinetic studies on the periodate oxidation of cellulose allowed the elaboration of a model that describes the reactions that occur simultaneously in the bulk and on the surface of fibers [131].

A modern and more complex method to assess the effects of periodate oxidation of cellulose consists of employing gel permeation chromatography (GPC) in association with multiple detection and carbonyl-selective fluorescence labeling according to the CCOA methodology profiling of carbonyl groups [132]. Applied to DAC, this analysis is limited by the concealing effect of bonds formed by hemiacetals moieties within one anhydroglugose unit or between two vicinal units, or even two units from neighboring macromolecular chains [133,134].

Solid-state ^13^C CP-MAS NMR spectroscopy was another method successfully employed to assess the degree of oxidation (DO) of cellulose oxidized in the presence of sodium periodate. The analysis took into consideration the quantification of the intricate signals of samples (washed after oxidation), when the sharp C1 signal of initial cellulose was used as an internal standard [135]. This method provided more data than the current traditional ways of assessing DO (UV-vis spectroscopic monitoring of periodate consumption and the oxime method), but only under specified experimental conditions. It did confirm, however, that the reaction followed a core-shell pattern, with an oxidized cellulose exterior layer covering an unaltered cellulose core.

A novel approach of evaluating DO has been developed and effectively tested recently. It is based on multivariate calibration and infrared spectroscopy (NIR and FTIR). [136], and the experimental data were obtained in very short time intervals (seconds). Sodium periodate consumption assessed by UV-vis spectroscopy or potentiometric titration were employed for calibration, but potentiometric titration was suggested for cross-validation.

Periodate oxidation of cellulose allowed the production of sterically stabilized nanocrystalline cellulose (SNCC) [137,138], when the size and morphology of nanofibers can be modulated by the means of oxidation conditions, and their increased thermal stability is due to the hemiacetal bonds that contributed to the crystallinity recovery.

**Scheme 5:**
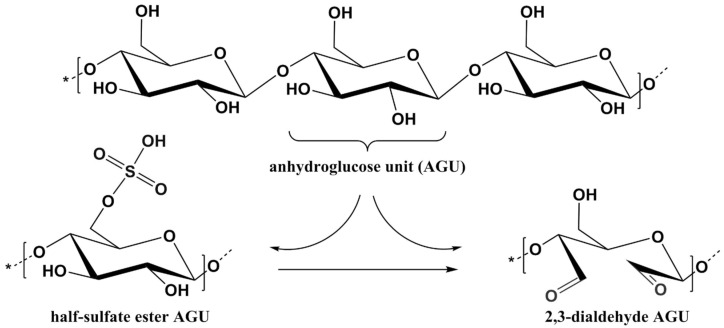
Structure of cellulose bearing sulfate half ester groups (C6) and DAC (C2, C3).

When applied to commercially available cellulose nanocrystals (CNCs) of industrial purity, the reactions that occur during periodate oxidation become more complex given the structural features of the substrate. It is generally known that these CNCs have a bulky substituent at C6, namely a sulfate half-ester group, which is formed during their preparation, when sulfuric acid hydrolysis and subsequent esterification stages are required. Since the periodate oxidation occurs at the C2 and C3 bonds, it appears that the substituent at C6 has no bearing on the oxidation process, and hence its impact was not studied. It has been proven that the degree of oxidation depended on the initial content in sulfate half-ester groups as they caused the decrease of the oxidation rate (the structures are illustrated in Scheme 5) [139].

On the other hand, the final products had a lower content of sulfur, suggesting that sulphate groups were also attacked during oxidation. These conclusions and dependencies were confirmed by experimental data, which also allowed the understanding of the mechanism of degradation of CNCs during periodate oxidation that entailed the loss of the surface layers.

### 2.4. Miscellaneous

Methods of selective oxidation of cellulose proved to be not only highly effective and regioselective, but also easy to combine with each other or with other methods in order to obtain cellulose with new and/or increased number of functional groups. The resulting products may have two carboxylic acid groups at C2 and C3 (as in DAC) or three carboxylic acid groups at C2, C3, and C6 (resulted from different one-pot one-step, two-step, or three-step procedures).

#### 2.4.1. The TEMPO-Periodate Approach

A very effective procedure of cellulose oxidation, when 2,3,6-tricarboxycellulose (TCC) (also known as mesotartaric acid/monohydrated glyoxilic acid alternating co-polyacetal) was obtained under moderate conditions, has been reported to evolve as one single process, namely the TEMPO-periodate reaction system [140]. This one-pot synthesis allowed highly water-soluble TCC in high yields and with high DO (in terms of carboxylic groups), a low degree of cellulose depolymerization, and, not to be neglected, with low production costs (reduced amounts of periodate; TEMPO reagent is 250 times cheaper than AZADO). The simultaneous presence of nitroxyl radicals and periodate anions granted the complete oxidation of primary (C6) and secondary (C2, C3) hydroxyl groups in anhydroglucose structural units. In a parallel experiment conducted for comparison reasons, another nitroxyl radical (PINO) was employed under the same conditions, but the results were rather poor.

This procedure has been employed to control certain characteristics of oxidized cellulose, such as transparency, rheological properties, microstructure [141,142], or ability to act as a crosslinking agent in high-performance materials [143,144].

It was also demonstrated that it was possible to modulate the degree of substitution by controlling the concentration of the periodate solution [64]. Thus, high concentrations of periodate entailed considerable degradation of the substrate (thus further promoting fibrillation and dissolution), while preserving the crystallinity and crystal width to a large extent.

#### 2.4.2. The Periodate-Chlorite Method

It has been already demonstrated that cellulose oxidation in the presence of periodate evolves with changes in the crystallinity and morphology of the substrate: the reduced crystallinity was associated with an increased degree of oxidation when the dialdehyde groups are not homogeneously distributed along the macromolecular chains [63], and the decreased aspect ratio was revealed by SEM [145]. When a new oxidation step was performed in the presence of sodium chlorite and hydrogen peroxide, at pH = 5, under stirring at room temperature for 24 h, the aldehyde groups were transformed into carboxylic ones [146] and the fractions separated after both stages of oxidation were analyzed. It was thus concluded that both oxidation reactions attacked preferentially the amorphous regions in cellulose (softwood cellulose pulp) causing a depolymerization and a subsequent dissolution, that were evidenced by the morphology (AFM, DLS) and crystallinity (XRD) study. This conclusion was also supported by FTIR data for the fraction with the highest content in carboxylic groups.

Another two-step procedure allowed cellulose to react first with sodium periodate for 5 h, in dark and under stirring, at 55 °C; then, after separation, DAC was further submitted to oxidation in the presence of sodium chlorite, for 24 h, at acidic pH (1 M acetic acid solution) [147]. Experimental data confirmed the almost complete conversion of aldehyde groups into carboxylic ones.

Viscose fibers submitted to this two-step oxidation method, but under mild conditions (low concentrations of oxidants, short reaction time intervals), were reported to achieve improved sorption properties and increased crystallinity compared to initial fibers [148].

This method was also employed to nanofibrillate [149], peel [150], or even disintegrate [151] cellulose from different sources. More recent studies have demonstrated that it was possible to tailor the surface properties of cellulose nanofibers by sequential oxidation using the periodate-chlorite reaction system [152,153] in order to prepare or even to recover high-performance materials.

#### 2.4.3. Other Methods

Although the classic method of TEMPO-mediated selective oxidation of cellulose represents yet the state-of-the-art in this field of research, sustained efforts are constantly made to improve it, particularly in terms of size of the obtained nanocellulose, yield and processability. In example, adding a post-oxidation ultrasonic-induced cellulose fragmentation process, it was possible to obtain carboxy-functionalized spherical cellulose nanoparticles from Alpha plant-derived cellulose microfibers (CMF: fiber diameter 7.26 μm, crystallinity 87%) [154]. The TEMPO-oxidized CMF (T-CMF: average diameter 2.72 μm, crystallinity 89%, content in carboxylic groups 1 mmol COO-/g cellulose) were subjected to ultrasonication for 45 min, when cellulose nanospheres resulted (CNS: average diameter 33.96 ± 7.01 nm, crystallinity 76%, content in carboxylic groups on the CNS surface ≈ 0.95 mmol COO-/g cellulose).

A complex, two-step experimental protocol has been designed to prepare TCC with high surface area and high surface charge. It is a combination of all three previously presented methods, namely TEMPO, periodate, and chlorite [155]. In the first stage, a TEMPO-assisted oxidation is performed on cellulose pulp from bagasse; then, the purified oxidation product was further submitted to periodate–chlorite oxidation when TCC with high content in carboxylic groups was obtained. Despite the decreased crystallinity, the material displayed good mechanical properties, a high transparency, and excellent sorption characteristics.

An environmentally friendly approach was reported to use a halogen-free 2,2,6,6-tetramethylpiperidine-1-oxyl (TEMPO)-mediated oxidation system that additionally employed lacasse and oxygen (TEMPO/laccase/O_2_-TLO system) for an oxidation under aqueous conditions [156]. It is well-known that selective oxidation of hardwood bleached kraft pulp (HBKP) under these conditions yields in cellulose nanofibers (CNFs) with a high content in carboxylic groups, but related research is still in its early stages. Using TLO system, it was possible to selectively oxidize cellulose from different sources (microcrystalline cellulose (MCC), softwood bleached kraft pulp (SBKP), bleached bagasse sulfate pulp (BBSP), and HBKP) with notable success regardless the origin of the substrate, thus proving the versatility of this oxidation system. Unlike standard TEMPO/NaBr/NaClO oxidation, when aldehyde groups formed on the surface of cellulose macromolecular chains were much less numerous (0.041–0.097 mmol/g) than the carboxylic moieties (0.846–1.154 mmol/g), the TLO oxidation system afforded lower content in carboxylic groups (0.558–0.615 mmol/g), but higher for aldehyde ones (0.218–0.235 mmol/g).

Highly crystalline cellulose nanofibrils have been successfully obtained using a novel and cost-effective oxidation system, namely ammonium persulfate (APS) under acidic conditions and long reaction intervals [157]. Thus, it was possible to modify the surface features of nanocellulose and, furthermore, to put together a set of conditions so that the synthesis is predictable and the parameters are reproducible, and, finally, nanofibers with specific properties are allowed. Optimization of the reaction system (amount of APS, reaction time, temperature regime) entailed changes in the nanocellulose properties: content in carboxylic groups 0.6–1.4 mmol/g; crystallinity index 72–88%; diameter 2.7–4.5 nm; length 146–247 nm; aspect ratio 48–80 nm. More notably, unlike the TEMPO-mediated oxidation, the ecologically friendly APS-assisted oxidation was effectively applied for the oxidation of lignin-containing feedstocks, used *per se* without previous treatments, when comparable results in terms of structural and chemical features have been obtained without modulating the experimental conditions.

Given the high demand for materials with diverse functional groups, combining several functionalization processes in a single method or in a cascade of events is of great interest. Such a complex approach has been designed to start with the selective oxidation of cellulose in the presence of sodium periodate, and then to continue *via* the green Passerini three-component reaction [158]. The use of periodate oxidation enhanced the substrate reactivity while preserving its fundamental features. The following processes allowed the grafting of two chemical precursors (a *tert*-butyl isocyanide and a carboxylic acid with an alkine or methacrylate moiety) onto oxidized cellulose through aldehyde groups. This method turned out to be a mild, versatile, and ecologically friendly synthetic strategy for the multiple functionalization of cellulose.

## 3. Environmental Applications of Materials Based on Selectively Oxidized Cellulose

### 3.1. General Considerations

Environmentally, the past few decades have proven to be highly challenging due to the aggressive industrialization, and to the increasing amounts of industrial water and biological hazard associated. Air, water, and soil have been contaminated with heavy metal salts and oxides, pesticides, dyes, drugs and pharmaceuticals, oil and organic solvents, etc. Many protocols have been developed for their decontamination and are now in use: biological treatments, flocculation, membrane/barrier processes, chemical precipitation, ion exchange, sorption onto activated carbon, etc. [37,159,160]. Still, these methods have some limitations (pH-sensitivity, moderate selectivity and removal capacity, high reagents and energy consumption, toxic by-products), so, the new approaches must take into consideration the bioremediation and biosorption [47,161,162]. The mechanism of adsorption consists, basically, of a mass transfer and accumulation at the interface of the phases in contact. Both physisorption (retention through weak and reversible linkages, such as hydrogen bonds and van der Waals forces) and chemisorption (strong chemical bonds formed between the sorbent surface and adsorbate) are involved in the removal of a compound from its solution when put in contact with a solid sorbent material.

An appropriate porosity and the presence of reactive functional groups are properties of high interest in sorbent materials, as both of them significantly contribute to the removal of pollutants. Thus, proton donor functional groups, such as carboxylic, hydroxylic, phenol, amine, amide, can successfully retain metal ions and organic molecules.

Therefore, the selective oxidation of cellulose from different renewable resources represents a valuable versatile method to obtain functionalized cellulose that can be successfully employed, *per se* or in complex formulations, as high-performance materials for environmental applications.

### 3.2. Main Characteristics of Selectively Oxidized Cellulose

Oxidized cellulose possesses a series of interesting characteristics, aside the afore mentioned ones, that make it suitable for biosorption and bioremediation. Thus, the morphology of oxidized cellulose (micro-, nano-) fibers and crystals, their swelling capacity, selective retention, thermal stability, mechanical properties and biodegradation have all a contribution to the level of performance of the material [62]. Tailoring wisely these properties enables a fine modulation of the material behavior.

Morphology studies (performed by various methods: optical microscopy, SEM, TEM, AFM) have all confirmed the defibrillation of the substrate associated with the oxidation, and the subsequent improved dispersity of cellulose fibrils bearing single or multiple functionality (aldehyde and/or carboxylic groups) [137,146,163,164,165]. For example, TEMPO-oxidized cellulose exhibited a web-like structure made of well-separated and randomly entangled fibers, evidenced by TEM [166,167]. The higher was the content in carboxylic groups, the smaller were the fibrils dimensions (nanometric scale). Still, it is yet to be discriminated if this particular morphology is due to the degree of oxidation, depolymerization, or the specificity of the source of cellulose.

The swelling capacity of oxidized cellulose fibers is significantly improved due to the presence of O-containing newly achieved functional groups that are able to link water molecules by H-bonds. The selective oxidation of different types of cellulose (non-fibrillated and partially fibrillated softwood, kraft fibers) allowed a homogeneous swelling without destroying the native crystalline structure of cellulose I [168]. TEMPO-oxidized fibers showed an increase in the pore volume upon swelling at different pH values [169]. Same results have been reported for oxidized fibers dispersed by ultrasonication, but with little effect on the bulk swelling [170].

Studies on the thermal stability of selectively oxidized cellulose indicated that these materials undergo thermal degradation reactions at lower temperatures than the initial cellulose [62,171], but they can be stabilized by further functionalization. Some films prepared from such oxidized cellulose, that consisted of randomly assembled nanofibers, exhibited extremely low coefficient of thermal expansion due to their high crystallinity [172]. Investigation on the effect of gradual oxidation of cellulose, that was yielding in cellulose bearing both aldehyde and carboxyl moieties at C2, C3, and C6 atoms, showed that 2,3-dialdehyde cellulose (DAC) was the most thermally stable product, while the carboxylate derivates were the most thermal-sensitive [173]. The same conclusion, that the presence of -COOH groups determined a lower onset of the thermal degradation temperature, has been reached for fibers used in nanopapers [174].

Mechanical properties of selectively oxidized cellulose fibers have been studied as well, taking into account that these reactions alter the crystallinity of the substrate and, hence, the tensile strength. Considering several oxidation protocols (TEMPO-mediated and TEMPO-periodate, oxidation in the presence of periodate, and chlorite oxidation), it was evidenced that periodate oxidation followed by borohydride reduction allowed more ductile fibers [175]. However, for films made of TEMPO-oxidized cellulose nanofibers that were bearing sodium carboxylate groups, it was found that the tensile strength and elastic modulus values were significantly higher than those of other materials (PVA, cellophane, PVA composites) [62]. Even more, the tensile strength can be further improved by turning -COONa groups into -COOH ones, able to form even more H-bonds between macromolecular chains.

Biodegradation of selectively oxidized cellulose is an imperative requirement when considering modern eco-friendly approaches in environmental decontamination and bioremediation. When materials with complex formulation are employed in eco-sensitive applications, their partial or complete degradation under environmental conditions after performing their role is highly desirable. Most of these chemical, photochemical, and/or enzyme-catalyzed reactions that lead to the degradation of oxidized cellulose may occur almost simultaneously due to the presence of water, microorganisms, and contaminants that may act as initiators and/or catalysts. Still, in order to understand the mechanism of biodegradation of oxidized cellulose in the presence of enzymes, experiments were conducted under controlled conditions [176,177,178], when it was possible to prove the complete biodegradation of the substrate.

### 3.3. Sorbents for the Removal of Heavy Metals

Water decontamination includes the removal of highly toxic, non-biodegradable heavy metals. Nowadays, there are several methods extensively used due to their high effectiveness: membrane filtration, precipitation, electrochemical treatment, and sorption onto adsorbent media. Cellulose-based sorbents are very attractive materials as they are efficient, low cost, available and renewable, easily convertible through functionalization, environmentally friendly [179]. Materials obtained by selective oxidation of cellulose are bearing numerous carboxyl groups able to bond high amounts of heavy metal cations, such as Co (II), Cd (II), Ni (II), Pb (II), Cr (VI) [47,60,180,181,182], as well as Cu (II), Fe (II), and As (V) [70,183,184,185]. This subject has attracted much interest and, therefore, it has been extensively studied as proven by the increasingly numerous reports available in literature.

One particular experimental approach has been studied in order to be applied for Pb(II) removal. Sequential oxidation of raw wood cellulose by a complex three-stage procedure (TEMPO-periodate-chlorite) has yielded in a cellulose derivative bearing three carboxylate groups (TCC) [167]. The sorbent material was able to retain high amounts of Pb(II) onto its surface (1569 mg/g), second only to nitro-oxidized cellulose nanofibers (their adsorption capacity of lead was 2270 mg/g), which is much less common for lead than for other heavy metals. It was concluded that Pb(II) was removed from its solution by an adsorption-precipitation mechanism.

Nanostructured sponges based on selectively oxidized cellulose are significantly efficient sorbents for heavy metal ions due to their particularly high porous structure and the presence of functional groups that are acting as active sites for metal ions coordination [2,162]. The high retention values were obtained upon the complete penetration of aqueous solution into the sorbent, despite the inhomogeneous distribution of ions. The concentration of metal ions decreased from the surface layers to the core of the adsorbent material, which was a clear indication that adsorption was limited by kinetic factors, namely the diffusion of solution through the sorbent.

Hydrogels, xerogels, and cryogels based on oxidized cellulose fibers have been also successfully used for the removal of heavy metals from wastewater [182,186,187,188,189]. Many other formulations have been designed to include oxidized cellulose fibers, such as PVA hybrid aerogels [190], alginate-based materials [183], graphene oxide-containing hybrid bio-nanocomposites [191], nanoparticles obtained by assembling PEI into TOCFN-PVA aerogels [185], sulfated and carboxylated cellulose nanofibrils [192]. All these materials are highly competitive in terms of adsorption capacity evaluated for numerous heavy metals. Some of these data are summarized in Table 1.

New concepts and new materials have been introduced as a result of constant concerns for the environment. Thus, high-performance nanostructured adsorbent materials have been prepared by so-called green procedures, using preponderantly TEMPO-oxidized cellulose nanofibers in formulations that contained lower-by-design amounts of synthetic polymers. These materials were intended for ecologically safe bio(nano)remediation of wastewater [1,161,193].

Another approach to build multifunctional sorbents has been recently reported. It refers to a one-step procedure to prepare all-cellulose membranes starting from cellulose fibers and cellulose microfibrils with different functional groups (pristine cellulose nanocrystals; TEMPO-oxidized moieties; a zwitterionic polymer-poly(cysteine methacrylate)-grafted onto cellulose nanocrystals), self-assembled at micro- and nanometer scale [194]. Given the presence and various nature of the reactive groups, the new membranes are able to retain ions of the heavy metals (Au III, Fe III, Co II), to catalytically inactivate dyes (such as Methylene Blue) by hydrogenation, and possess antifouling and antibacterial properties.

A recent life cycle assessment (LCA) study confirmed that the selective oxidation of cellulose, as a part of the more complex manufacturing process, had a very low environmental impact [195]. This behavior contributed to the overall reduction of the ecological impact of the entire technological process.

### 3.4. Adsorbent Materials for Dyes

Persistent organic pollutants, such as dyes, are difficult to remove from water. Since their chemical structure consists of high amounts of aromatics, halogens, and metal ions, they can cause serious damages upon their careless discharge in water streams. They interact with the entire aquatic ecosystem and increase the water turbidity, which entails negative effects: reduced oxygenation, altered photosynthesis, diminished self-cleaning capacity [37]. According to their structure, dyes can be basic (cationic) (methylene blue, basic fuchsin, crystal violet, etc.), acid (Congo Red, eosin, etc.), non-ionic (disperse), direct (substantive), reactive, mordants, etc.

Bio-based nanostructured materials are a modern solution for the dyes removal from wastewater as they provide high surface area and reactivity, thus effectively contributing to the sustainability of bio-remediation. Following this research line, recent studies reported on the systematic use of naturally-derived nanomaterials for the design and preparation of porous nano-structured sorbents. Thus, functionalized polysaccharides are ideal candidates, especially when it comes to TEMPO-oxidized cellulose nanofibers (TOCNFs). They have been used for the highly efficient removal of Methylene Blue [196,197], Brilliant Blue [198], Basic Violet 1 and Rhodamine 6 g [199], or Toluidine Blue [200].

Highly porous aerogels based on oxidized cellulose were also employed in dyes removal, namely Methylene Blue and Rhodamine B [201]. These materials exhibited strong antioxidant activity due to the presence of TiO_2_ in their formulation, and enhanced sorption capacity.

When TOCNFs were crosslinked with branched polyethyleneimine, nanostructured sorbents were obtained. Their multiple functionalities make them able to react with metal ions and organic molecules, but mainly with dyes [161,202,203]. Their sorption efficiency was satisfactory under controlled pH conditions, and this feature was explained by the electrostatic interactions between dye molecules and amine groups in the structure of the synthetic polymer crosslinked with oxidized cellulose.

Nanostructured sponge-like sorbents have been prepared as well [204] and tested on commercially available dyes bearing different numbers of sodium sulfonate moieties. The study concluded that sorption process was pH-sensitive, the sorbent-sorbate interactions were complex (electrostatic attraction forces, as well as other intermolecular bonds) and depended on the number of sulfonate groups. Moreover, the sorbent can be regenerated/cleaned up and ready to be reused in a new decontamination cycle with the same efficiency.

### 3.5. Other Organic Pollutants

Organic solvents and oil are other pollutants often found in the environment and their strong negative impact is well documented. Despite the wide variety of materials used as practical solutions on large scale, new methods and materials are still needed.

Silane-coated PVA hybrid aerogels containing oxidized cellulose nanofibers have showed remarkable sorption capacity for crude oil and organic solvents, such as hexane [190]. Nano-structured sponges based on crosslinked PEI and TOCNFs have been successfully tested for the removal of *p*-nitrophenol from water [161], their capacity being much higher than that of other microporous sorbents. Aerogels based on TOCNFs have been prepared [205] and their capacity to retain oil and organic solvents was evaluated in comparison with other known sorbents. The materials showed good sorption capacity and selectivity owing to their excellent porosity and hydrophobic properties, they were able to undergo recycling while preserving their dye removal ability, and were fire-resistant. Last but not least, their production cost was rather low.

Other organic pollutants are herbicides and insecticides used in agriculture. In example, 1,1-dimethyl-4,4′-dipyridinium chloride (also known as paraquat) is extensively used as herbicide, despite its high toxicity, which led to contamination of both surface and underground waters. Therefore, new TEMPO-oxidized cellulose nanofibers were employed to retain paraquat from wastewater [92] and it was demonstrated that their efficiency strongly depended on the functionality and diameter of nanofibers, while preserving their initial crystallinity, and the adsorption of paraquat was significant for pH ≥ 7.

Other organic molecules, such as proteins [206] and enzymes [207] were adsorbed and stabilized onto TOCNFs. Proteins with different surface charge (lysozyme and bovine albumin serum) were retained by electrostatic forces and preserved their secondary structures. Selected enzymes were adsorbed and immobilized by electrostatic forces as well, but their activity was enhanced after adsorption and then maintained for a long period.

### 3.6. Other Applications

Nanocomposite materials based on TOCNFs and graphene nanosheets (GN) have been prepared and intended for alcohol sensing devices [208]. They exhibited high ethanol selectivity, remarkably higher than the sensor made of GN exclusively, a rapid response, and a broad range of detected alcohols. Conductive composite films of TOCNFs and polypyrrole (PPy) have been prepared, and their good mechanical properties and high conductivity recommended them as fit for sensors [209].

Novel pH-sensitive materials based on TEMPO-oxidized cellulose were obtained by further functionalization of TOCNFs as to obtain cellulose bearing ester and amine functional groups, respectively [210]. Their sensing ability has been monitored by color changes associated to different pH values.

A remarkable application of TEMPO-oxidized cellulose is the synthesis of new peptide conjugates able to detect the human neutrophil elastase with enhanced sensitivity [211]. Compared with other nano-structured cellulosic materials, these conjugates showed remarkable sensing ability due to their high porosity and small volume of crystallites which allowed the incorporation of large amounts of peptide.

## 4. Conclusions and Future Trends

This study presented some of the most recent advances in the field of materials based on selectively oxidized cellulose used in environmental applications.

The first and foremost conclusion is that this field of research is still highly active, and new strategies and materials are continuously emerging. Unfortunately, most of the reported experimental data remained just as literature, as only few of them were transferred to practice.

Selective oxidation of cellulose is widely and extensively used as a multitask platform to obtain key-materials having multiple functionalities. As it has been proved by the impressive volume of information reported in the literature, this is a highly effective method of functionalization, able to be finely tuned in order to yield in oxidized cellulose with modulated properties. Cellulose in various forms, raw or previously refined, and from various renewable and easily available resources has been successfully submitted to this derivatization procedure. The bio-based micro- and nanostructured materials thus produced were used in environmental applications, either *per se* or in complex multicomponent formulations (composite and hybrid materials).

The removal of heavy metals and main organic pollutants by adsorption onto sorbents based on selectively oxidized cellulose can be achieved by different approaches. This study reviewed some of the most recent literature data and illustrated the variety of procedures and materials. New concepts, such as bio-remediation, and novel green experimental protocols have been developed. Even the possibility to use the selectively oxidized cellulose for sensors has been explored.

Industrial transfer of these methods and materials requires further research focused on the possibility to regenerate and recycle the sorbents without causing secondary pollution, the implementation of novel green processes, and even on the manufacturing of new sorbent materials with multiple functionality able to simultaneously retain different pollutants. At the same time, the cost-effectiveness of these materials and methods must be addressed as well.

It is therefore possible to reduce the negative environmental impact of various pollutants by submitting low cost, easily available raw materials from renewable resources to functionalization through classic chemical reactions. Nevertheless, the constant efforts of academics and industrial R&D scientists need also the support of the entire society by environmentally-driven wise political and economic decisions, a satisfactory level of funding from public and private sources, and the honest and diligent commitment of the industrial end-users to apply the results of research.

## Figures and Tables

**Table 1:** Retention of some heavy metal ions onto oxidized cellulose-based sorbent materials (modified from reference [47]).

Metal Ion	Sorbent	Qmax (mg/g)	Observations
Cu(II)	TOCNFs	75	Cu(II) was reduced to self-assembled Cu(0) nanoparticles
	TOCNFs membranes	374	membranes with high water permeability, mechanical stability, and functionality
	TOCNFs hydrogel	268.2	pH = 5.0–6.0
	TOCNFs aerogel	303	
	TOCNFs-PEI	52.32	pH = 5.0, T = 30 °C, t = 20 h
	TOCNFs-PVA hybrid aerogel	151.3	
	TOCNFs-alginate	105.2–204.1	
	TOCNFs-GO	63.5–68.1	
Cd(II)	TOCNFs	140.3	pH = 5.5
	TOCNFs hydrogel	115	
Ni(II)	TOCNFs	49	pH = 6
Zn(II)	TOCNFs	66	pH = 6
Pb(II)	TOCNFs	137.7	pH = 5.0
	TOCNFs-PVA hybrid aerogel	110.6	From solution of mixed heavy metal sals
Hg(II)	TOCNFs-PVA hybrid aerogel	157.5	From solution of mixed heavy metal sals
Fe(II)	*In situ* modified TOCNFs membranes	456	redox system Fe(II)/Fe(III)
Cr(III)	TOCNFs	58	pH = 5.0
Cr(VI)	TOCNFs-PAN membrane	87.5	pH = 4.0, bichromate solution
Cs(I)	TOCNFs hydrogel	133.8	
Au(III)	TOCNFs hydrogel	15.44	pH = 2.0, 48 h

Notations: Qmax—adsorption capacity; TOCNFs—TEMPO-oxidized cellulose nanofibers; PEI—polyethylenimine; PAN—polyacrylonitrile; PVA—poly(vinyl alcohol); GO—graphene oxide.
